# Chlorthalidone, not hydrochlorothiazide, is the right diuretic for comparison

**DOI:** 10.1186/s40885-018-0089-1

**Published:** 2018-03-01

**Authors:** Ravi Tejraj Mehta, Anil Pareek, Indranil Purkait

**Affiliations:** Ipca Laboratories Limited, Mumbai, India

**Keywords:** Chlorthalidone, Hydrochlorothiazide, Amlodipine, Hypertension, Thiazide diuretics, Calcium channel blockers

## Abstract

We have read the study design “Comparison of effects between calcium channel blocker and diuretics in combination with angiotensin II receptor blocker on 24-h central blood pressure and vascular hemodynamic parameters in hypertensive patients: study design for a multicenter, double-blinded, active controlled, phase 4, randomized trial” by Oh GC, et al. with interest. The authors aim to compare the efficacy of amlodipine or hydrochlorothiazide (HCTZ) with an ARB. However, we wish to highlight that chlorthalidone (CTD) is the evidence-based and recommended anti-hypertensive diuretic, and should replace HCTZ in the trial to effectively compare efficacy against the CCB amlodipine.

Dear Editor.

We have read with interest the study design by Oh GC, et al. [1]. The authors aim to compare the efficacy of combination of calcium channel blocker (CCB) or thiazide diuretic with an angiotensin receptor blocker (ARB). However, we wish to highlight that instead of hydrochlorothiazide (HCTZ), chlorthalidone (CTD) is the evidence-based anti-hypertensive diuretic and should be used to effectively compare efficacy against the CCB amlodipine.

1. CTD is structurally and pharmacokinetically distinct from HCTZ with a much longer half-life (40–60 h vs. 3.2–13.1 h) and a wider volume of distribution. Hence, comparing HCTZ to amlodipine (half-life 38-50 h), with such disparity in half-lives, can lead to unsurprising differences in favour of amlodipine. CTD has unique pleiotropic properties, not shared by HCTZ, like inhibition of platelet aggregation and promotion of angiogenesis [2]. These features impart CTD a unique advantage to effectively lower BP as well as improve cardiovascular (CV) outcomes in hypertension [3].
2. CTD has been employed in several major National Institutes of Health (NIH)-funded randomized controlled trials evaluating hard outcomes: the Hypertension Detection and Follow-Up Program (HDFP), Multiple Risk Factor Intervention Trial (MRFIT), Systolic Hypertension in the Elderly Program (SHEP) and the Antihypertensive and Lipid-Lowering Treatment to Prevent Heart Attack Trial (ALLHAT); and has been repeatedly shown to reduce CV morbidity and mortality at clinically used doses. On the other hand, HCTZ at the usual prescribed doses (12.5-25 mg/day) has been called a “paltry” antihypertensive, inferior to all other drug classes, and with no published evidence of reducing CV events [4]. Major global hypertension guidelines including latest ones by ACC/AHA [5], CHEP [6] and NICE [7] recommend preferring chlorthalidone (thiazide-like diuretics) over HCTZ and bendroflumethiazide.
3. The authors have listed measurements of ambulatory pulse wave velocity (PWV), and augmentation index (AIx) as secondary endpoints. In a recent randomized, prospective cross-over study, effects of CTD 12.5 mg vs. HCTZ 25 mg, both in combination with ARB (Candesartan 8 mg), on PWV and AIx were compared. After 8 weeks, CTD arm showed a significant reduction in PWV vs. baseline (*p* = 0.007) and HCTZ (*p* = 0.033) [8].
4. In the MRFIT, in the nine clinics where HCTZ was prescribed predominantly initially, there was a 44% higher CHD mortality, whereas the trend of mortality was favorable in the six clinics that primarily used CTD. It was recommended and accepted by the MRFIT Steering Committee to switch all participants from HCTZ to CTD. Later, with CTD, the mortality trend was reversed and the same group had a 28% lower risk [9]. Similarly, it has been suggested that the findings of Avoiding Cardiovascular Events Through Combination Therapy in Patients Living With Systolic Hypertension (ACCOMPLISH) trial would have been much more compelling had CTD been the agent selected for the diuretic-based regimen [10].
5. In a randomized, multicenter trial, we found losartan/CTD combination to be as effective as losartan/HCTZ in lowering office BP and was well tolerated [11]. In another recent study, we compared CTD with HCTZ by 24-h ambulatory BP monitoring and found that CTD significantly reduced 24-h ABP as well as daytime and nighttime BP. However, no significant 24-h ABP reduction was seen with HCTZ, which merely converted sustained hypertension into masked hypertension [12].
6. CCBs have been shown to be better at reducing central BP and arterial stiffness than thiazide-type diuretics leading the authors to hypothesize that CCB-based combination will be associated with better CV outcomes. However, in ALLHAT study, substantially higher risk of heart failure with amlodipine was found compared to CTD (RR 1.38; 95% CI, 1.25-1.52); risk being even higher in patients with diabetes (RR 1.42; 95% CI, 1.23-1.64) [13]. Thus, the CV outcome of the two combinations must be assessed in a long-term study.

To summarize, we urge the authors to re-think on the choice of HCTZ as the comparator diuretic and replace it with CTD, if feasible.

## Abbreviations

ACC/AHA: American College of Cardiology/American Heart Association
AIx: Augmentation index
ALLHAT: Antihypertensive and Lipid-Lowering Treatment to Prevent Heart Attack Trial
ARB: Angiotensin receptor blocker
CCB: Calcium channel blocker
CHEP: Canadian Hypertension Education Program
CTD: Chlorthalidone
HCTZ: Hydrochlorothiazide
HDFP: Hypertension Detection and Follow-Up Program
MRFIT: Multiple Risk Factor Intervention Trial
NICE: National Institute for Health and Care Excellence
NIH: National Institutes of Health
PWV: Pulse wave velocity
SHEP: Systolic Hypertension in the Elderly Program
